# Automated fracture screening using an object detection algorithm on whole-body trauma computed tomography

**DOI:** 10.1038/s41598-022-20996-w

**Published:** 2022-10-03

**Authors:** Takaki Inoue, Satoshi Maki, Takeo Furuya, Yukio Mikami, Masaya Mizutani, Ikko Takada, Sho Okimatsu, Atsushi Yunde, Masataka Miura, Yuki Shiratani, Yuki Nagashima, Juntaro Maruyama, Yasuhiro Shiga, Kazuhide Inage, Sumihisa Orita, Yawara Eguchi, Seiji Ohtori

**Affiliations:** 1 Department of Orthopaedic Surgery, Chiba University Graduate School of Medicine, 1-8-1 Inohana, Chuou-Ku, Chiba, 260-8670 Japan; 2 Center for Frontier Medical Engineering, Chiba University, Chiba, Japan

**Keywords:** Preclinical research, Medical imaging

## Abstract

The emergency department is an environment with a potential risk for diagnostic errors during trauma care, particularly for fractures. Convolutional neural network (CNN) deep learning methods are now widely used in medicine because they improve diagnostic accuracy, decrease misinterpretation, and improve efficiency. In this study, we investigated whether automatic localization and classification using CNN could be applied to pelvic, rib, and spine fractures. We also examined whether this fracture detection algorithm could help physicians in fracture diagnosis. A total of 7664 whole-body CT axial slices (chest, abdomen, pelvis) from 200 patients were used. Sensitivity, precision, and F1-score were calculated to evaluate the performance of the CNN model. For the grouped mean values for pelvic, spine, or rib fractures, the sensitivity was 0.786, precision was 0.648, and F1-score was 0.711. Moreover, with CNN model assistance, surgeons showed improved sensitivity for detecting fractures and the time of reading and interpreting CT scans was reduced, especially for less experienced orthopedic surgeons. Application of the CNN model may lead to reductions in missed fractures from whole-body CT images and to faster workflows and improved patient care through efficient diagnosis in polytrauma patients.

## Introduction

The emergency department is an environment with a potential risk for diagnostic errors during trauma care, particularly for fractures. Diagnostic errors and serious complications occur in patients with polytrauma for a number of reasons, including insufficient medical history, severity and complexity of the injury, and because the clinician is responsible for multiple simultaneous tasks. Furthermore, some of medical staff working at Level 1 trauma centers may be less experienced in managing polytrauma patients^1–3^. Another major reason that misinterpretations frequently occur is due to the crucial need for a rapid diagnosis to start treatment, and an extended interpretation time is required for the large number of whole-body computed tomography (CT) images that are obtained for polytrauma patients^1^. For such patients, misinterpretation of fractures may result in delayed treatment and poor outcomes^4^. Therefore, physicians in the emergency department must assess patients with polytrauma in a systematic, concentrated, accurate and rapid manner.

In the primary management of patients with multiple traumas, whole-body CT scanning is recommended as a standard of care^5^. It is well established that CT scanning is superior to plain radiographs in the evaluation of fractures. Nonetheless, missed diagnoses are common and in whole-body CT performed overnight and on weekends for patients with multiple trauma, 12.9% of injuries were overlooked at the initial interpretation^6^. For fractures specifically, the rate of a missed lumbar spine fracture diagnosis on abdominal and pelvic CT images was 23.2% at a Level 1 trauma center^7^. In another study, 20.7% of rib fractures were missed on initial chest CT evaluation^8^. Therefore, there is a great demand to improve the accuracy of clinical diagnoses and reduce the incidence of missed fractures^6^.

Convolutional neural network (CNN) deep learning methods are now widely used in medicine because they improve diagnostic accuracy, decrease misinterpretation, and improve efficiency^9,10^. Indeed, the number of clinical uses for CNN in the fields of orthopedic surgery and traumatology is increasing^11–14^. New CNN algorithms reduce human workload and can extract features that are difficult for humans to recognize. Some studies have reported fracture identification using CNN on radiographs and CT scans^15–17^. However, to our knowledge, automatic localization and classification of fractures in CT using CNN methods have only been reported for rib and pelvic fractures and each model can detect only a single type of fracture^16,17^.

The emergency department is an environment in which fractures of the pelvis, ribs, and spine can be missed, leading to delayed treatment and poor prognosis. CNN models might be a great potential to reduce these missed fractures. In this study, we investigated whether automatic localization and classification using CNN could be applied to pelvic, rib, and spine fractures. We also examined whether this fracture detection algorithm could help orthopedic surgeons in fracture diagnosis by comparing the sensitivity, precision, and diagnostic time of the orthopedic surgeons with and without CNN model assistance.

## Materials and methods

### Patients

The study protocol was approved by the local institutional review board of the Graduate School of Medicine, Chiba University. (Reference number 3329) The requirement for informed consent was waived by the local institutional review board of the Graduate School of Medicine, Chiba University because of the retrospective analysis. All procedures for human participants adhered to the 1964 Declaration of Helsinki and subsequent amendments. All images were collected anonymously to ensure that no private information (such as patient name, gender, and age) was revealed.

**Figure 1:**
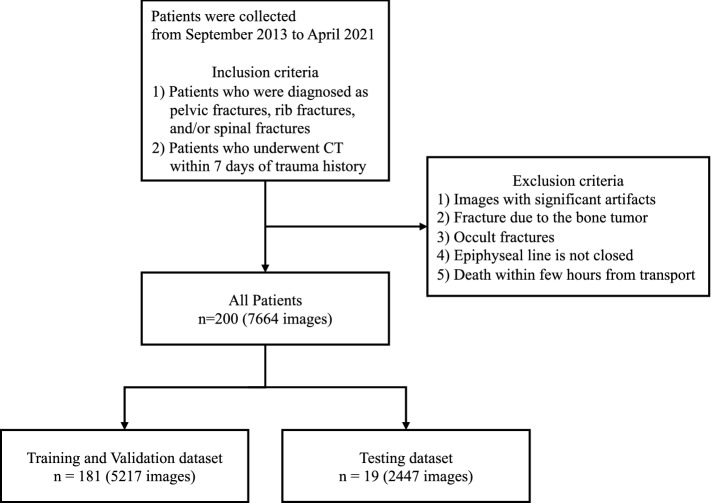
Flow chart showing the overall study process. CT, computed tomography.

We retrospectively reviewed the medical records of patients who had been transported to the emergency room for trauma at our hospital between September 2013 and April 2021. The inclusion criteria were as follows: (1) patients who were diagnosed with pelvic fractures, rib fractures, and/or spine fractures, (2) patients who underwent CT (either plain or contrast-enhanced examination) within 7 days of trauma. The exclusion criteria were as follows: (1) images with significant artifacts affecting the diagnosis, (2) fractures due to a metastatic bone tumor, (3) occult fractures that can only be confirmed by MRI, (4) patients before epiphyseal closure, (5) patients who died within a few hours of transport. In this study, fractures of the cervical vertebrae were not included because of their unique shape compared to the thoracolumbar spine and their smaller size compared to the slice thickness of the CT scan. In addition, this study did not include clavicle, scapula, humerus, sternal, and femoral fractures due to the small proportion of these fractures present in this study cohort. The analysis included a total of 200 patients. Figure 1 shows the flow chart of the study process.

### Datasets

A total of 7664 whole-body CT axial slices (chest, abdomen, pelvis) from 200 patients were used. CT scans were performed according to our standard protocol for whole-body trauma CT: the scan extended from the skull vertex to the upper thighs. The peak kilovoltage was 120 kVp, and an automatic tube current modulation was performed. The CT axial slices used in this study were bone (width, 1900–2000; level, 200–500) window images, in which the slice thickness was 0.5 to 5 mm. CT from digital imaging and communications in medicine (DICOM) files were exported in “JPG” format from the picture archiving and communication systems in our hospital. Each CT axial slice was digitized, saved as a "JPG" format image file with a specific identification code in a size of approximately 512 × 512 pixels, and removed the black background and trimmed to include the area of the patient's trunk. We used 189 patients for the training and validation datasets and selected 19 patients for the testing dataset. CT axial slices from patients were divided into training, validation, and testing datasets for analysis. The CNN was trained on 4174 axial slices, with 1043 axial slices held out for validation and 2447 axial slices for testing. Training and validation datasets contained only annotated images and no fracture-free images. The testing dataset encompassed a series of CT axial slices (5-mm slice thickness) of the patient's entire chest to pelvis, including unfractured images.

### Imaging annotation and preprocessing

**Figure 2:**
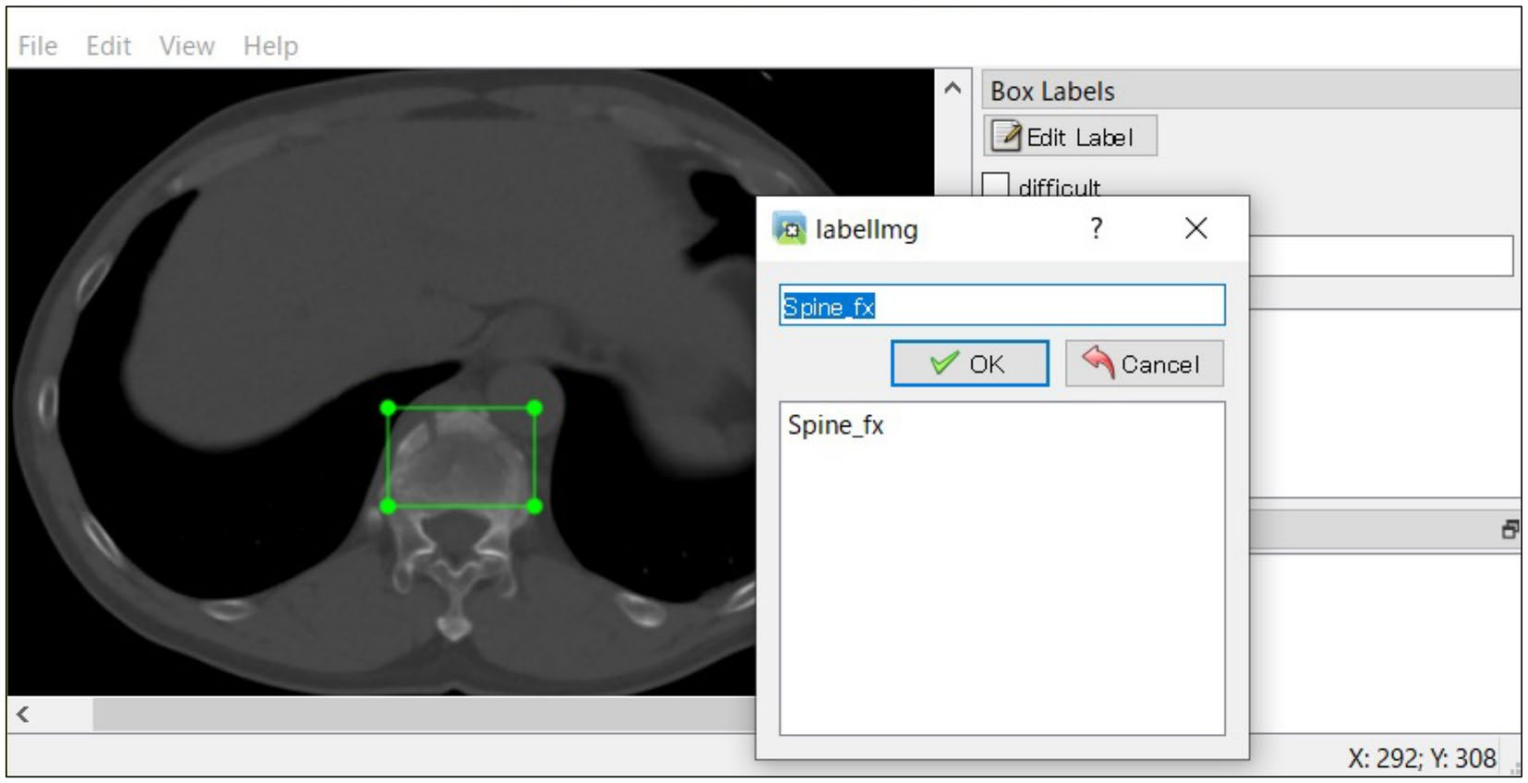
A screenshot of the image annotation process. We removed the black background and trimmed to include the area of the patient's trunk. A rectangular bounding box covering the minimum area of the fracture site was then drawn on every fracture of the CT slice and was labeled as spine fractures, pelvic fractures, or rib fractures using labelImg (version: 1.8.1, available at https://github.com/tzutalin/labelImg). CT, computed tomography.

The annotation process is shown in Fig. 2. The ground truth was determined by two experienced orthopedic surgeons. Namely, the CT axial slices with fractures were annotated by a board-certified orthopedic surgeon (TI, 8 years of experience) and checked by a senior board-certified orthopedic surgeon (SM, 15 years of experience). If the conclusion was inconsistent between the two surgeons, we referred to the radiology report and confirmed images in coronal and sagittal planes of the fracture.

### CNN model construction

**Figure 3:**
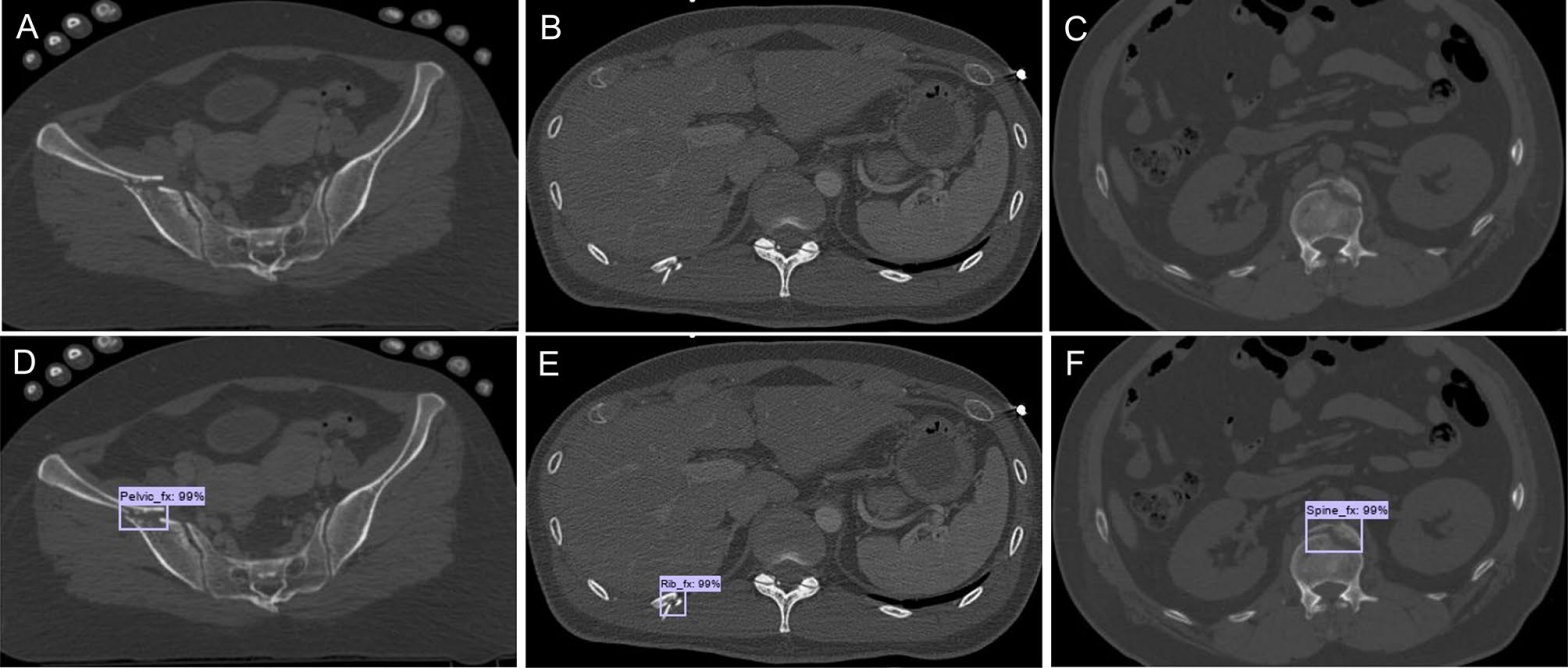
Sample CT images correctly annotated by neural networks on the testing dataset. (**A** and **D**) Pelvic fracture. (**B** and **E**) Rib fracture. (**C** and **F**) Spine fracture. The CNN model also generated a confidence score for each of the detected points as continuous values of a range from 0 to 100%). CT, computed tomography; CNN, convolutional neural network.

For the neural network model, we selected an object detection tool package based on TensorFlow (https://github.com/tensorflow/models/tree/master/research/object_detection), which is one of the most advanced object detection algorithms for multiple categories. Faster R-CNN^18^, a state-of-the-art object detection framework, is at the core of the model in this tool package, and the base CNN used in our model was a Faster-RCNN-Inception-V2-COCO. Faster R-CNN is based on a CNN with additional components for detecting, localizing, and classifying objects in an image. A set of 5217 annotated CT axial slices was used to train the object recognition Faster R-CNN. The training parameters were assigned as follows: a batch size of 1; a total of 200,000 iterations; and an initial learning rate of 0.0002. The training process was executed on a GPU (GeForce RTX 2060, NVIDIA, USA). The Python programming language, version 3.6.7 (https://www.python.org) and Google’s open source deep learning framework Tensorflow, version 1.14.0 (https://www.tensorflow.org) were used to construct the CNN architecture. The operating system was Windows 10. Figure 3 shows a sample image of the testing dataset annotated correctly by the neural network.

### Performance evaluation

In our study, we investigated the performance of the CNN model itself and compared diagnostic capabilities among orthopedists with or without assistance by the CNN model. To objectively evaluate the performance of the Faster R-CNN, three evaluation indicators (sensitivity, precision, F1-score) were calculated as follows: sensitivity = true positive/(true positive + false negative); precision = true positive/(true positive + false positive); and F1-score = 2 × sensitivity × precision/(sensitivity + precision). True positive denotes fractures that were correctly identified both by classification and location. False positives indicate fractures whose classification or location was not correctly identified or whose anatomically normal structures had been detected as fractures. If fractures were not detected, the examination was classified as a false negative. It was not possible to define a true negative due to the nature of this task, as multiple objects could be detected for every image. To compare diagnostic capabilities among orthopedic surgeons with or without assistance by the CNN model, sensitivity, precision, and diagnosis time were judged for each image. Firstly, three orthopedic surgeons (with 3, 3, and 8 years of experience, respectively) reviewed the CT axial slices without the support of the CNN. Example ground truth annotations in the images of the training and validation datasets were shown to the orthopedists, from which they learned how to annotate. The orthopedists were required to read and record the localization of the fracture using the bone window with whole-body CT axial slices. The second test was conducted two months later with the support of the CNN. The diagnosis time for the first and second tests was recorded using a stopwatch.

### Statistical analysis

We conducted statistical analyses using JMP (version 15, SAS Institute). The sensitivity of the orthopedic surgeons’ diagnostic performance with or without assistance from the CNN model was compared using a McNemar test. The orthopedic surgeon’s time to diagnosis with or without CNN model assistance was compared using paired *t* test. A *p* value < 0.05 denoted a significant difference. It was not possible to compare the precision because the number and location of false positives was inconsistent between the orthopedic surgeons with and without the support of the CNN model.

## Results

### Patient characteristics

The mean age of the 200 patients included in this study was 54.0 ± 20.6, and the male:female ratio was 130:70. Demographic data of the patients included in the training and validation datasets are presented in Table 1. The demographic data of the patients included in the testing dataset are shown in Table 2. A total of 2447 images were used in the testing dataset, with an average of 129 images per patient. Among all the images used in testing dataset, we included 143 pelvic fractures, 87 rib fractures, and 136 spine fractures. The prevalence of each fracture among all the images used in testing dataset was 5.8% for pelvic fractures, 3.6% for rib fractures, and 5.5% for spine fractures.

**Table 1:** Demographic data of patients included in the training and validation dataset.

	Training and validation dataset
No. of patients	181
Age, mean ± SD	54.3 ± 20.8
Sex (male: female)	117:64
No. of CT images	5217
No. of annotations	6357
Pelvic fracture	2014
Rib fracture	1897
Spine fracture	2446

Data are shown as n and mean ± standard deviation.

*CT* computed tomography, *No.* number, *SD* standard deviation.

**Table 2:** Demographic data of patients included in the testing dataset.

	Testing dataset
No. of patients	19
Age, mean ± SD	51.2 ± 19.2
Sex (male:female)	13:6
No. of CT images	2447
**No. of fractures (per view)**	
Pelvic fracture	143
Rib fracture	87
Spine fracture	136
**Prevalence (%)**	
Pelvic fracture	5.8
Rib fracture	3.6
Spine fracture	5.5

Data are shown as n and mean ± standard deviation.

*CT* computed tomography, *No.* number, *SD* standard deviation.

### Performance of the CNN model

**Figure 4:**
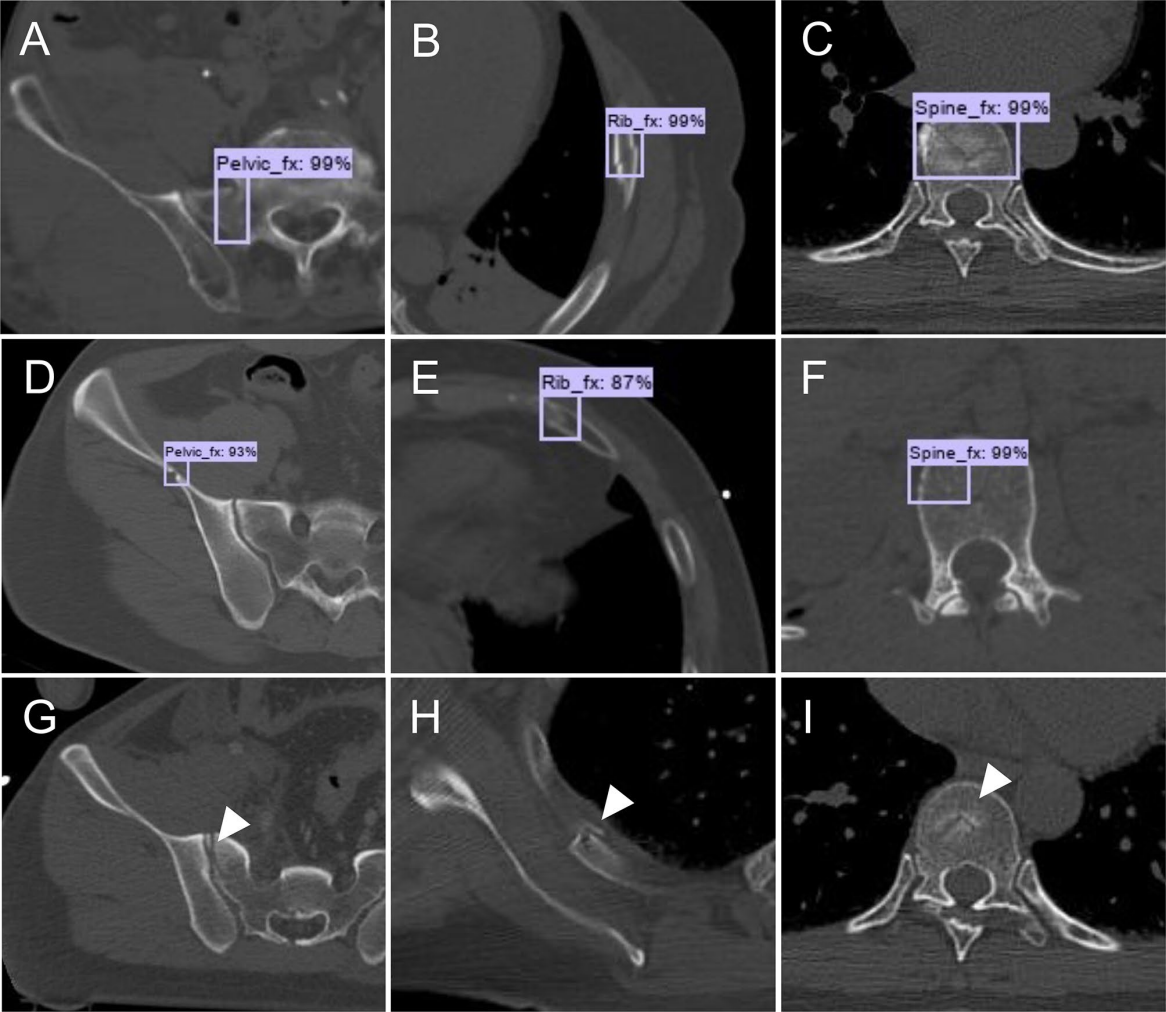
Representative images of true positive, false positive, and false negative. (**A**–**C**) CT axial slices detected and diagnosed correctly by the CNN model for three fractures (true positive). (**D**–**F**) CT axial slices of a misdiagnosed fracture (false positive). The vascular groove was mistaken for a pelvic fracture (**D**); The boundary between costal cartilage and rib was mistaken for a rib fracture (**E**); The vascular groove was mistaken for a spine fracture (**F**). (**G**–**I**) CT axial slices of a missed fracture (false negative were indicated by arrowheads). CT, computed tomography; CNN, convolutional neural network.

The sensitivity, precision, and F1-score of the CNN model are shown in Table 3. For pelvic fractures, the mean sensitivity was 0.839, the mean precision was 0.645, and the mean F1-score was 0.729. For rib fractures, the mean sensitivity was 0.713, the mean precision was 0.602, and the mean F1-score was 0.652. For spine fractures, the mean sensitivity was 0.780, the mean precision was 0.683, and the mean F1-score was 0.729. For the grouped mean values for all three types of fractures, the sensitivity was 0.786, precision was 0.648, and F1-score was 0.711. Representative images of true positive, false positive, and false negative are shown in Fig. 4.

**Table 3:** Performance of the CNN model.

	Pelvic fracture	Rib fracture	Spine fracture	Mean
**CNN**				
Sensitivity (%)	0.839	0.713	0.780	0.786
Precision (%)	0.645	0.602	0.683	0.648
F1-score (%)	0.729	0.652	0.729	0.711

*CNN* convolutional neural network.

### Comparison of diagnostic capabilities of orthopedic surgeons with and without CNN model assistance

The sensitivity and precision of the diagnostic ability of the orthopedic surgeons with and without CNN model assistance are shown in Tables 4 and 5. The sensitivity of orthopedic surgeons increased after CNN model assistance for pelvic fractures, rib fractures, and spine fractures. This was particularly the case for the less experienced orthopedic surgeons (orthopedic surgeons 1 and 2), where the sensitivity was statistically significantly increased after using the CNN model. In contrast, the precision remained the same with or without CNN assistance. In comparing the diagnostic time of orthopedic surgeons with and without CNN, the time was significantly reduced when using the CNN model (Table 6).

**Table 4:** Comparison of sensitivity for orthopedic surgeons with and without CNN model assistance.

Sensitivity (%)	Without CNN model assistance	With CNN model assistance	*p* value
**Pelvic fracture**			
Orthopedic surgeon 1	0.822	0.892	0.0158*
Orthopedic surgeon 2	0.808	0.893	0.0060*
Orthopedic surgeon 3	0.853	0.881	0.0184*
**Rib fracture**			
Orthopedic surgeon 1	0.580	0.747	0.0006*
Orthopedic surgeon 2	0.596	0.729	0.0093*
Orthopedic surgeon 3	0.651	0.719	0.1573
**Spine fracture**			
Orthopedic surgeon 1	0.649	0.814	0.0023*
Orthopedic surgeon 2	0.676	0.795	< 0.0001*
Orthopedic surgeon 3	0.762	0.838	0.0143*
**Mean**			
Orthopedic surgeon 1	0.697	0.829	< 0.0001*
Orthopedic surgeon 2	0.676	0.818	< 0.0001*
Orthopedic surgeon 3	0.764	0.826	0.0003*

Orthopedic surgeon 1 and 2 had 3 years of experience. Orthopedic surgeon 3 had 8 years of experience. CNN, convolutional neural network.

*p < 0.05.

**Table 5:** Comparison of precision for orthopedic surgeons with and without CNN model assistance.

Precision (%)	Without CNN model assistance	With CNN model assistance
**Pelvic fracture**		
Orthopedic surgeon 1	0.957	0.976
Orthopedic surgeon 2	0.974	0.976
Orthopedic surgeon 3	0.961	0.947
**Rib fracture**		
Orthopedic surgeon 1	0.962	0.942
Orthopedic surgeon 2	0.949	0.885
Orthopedic surgeon 3	0.935	0.984
**Spine fracture**		
Orthopedic surgeon 1	0.977	0.940
Orthopedic surgeon 2	0.952	0.956
Orthopedic surgeon 3	0.961	0.950
**Mean**		
Orthopedic surgeon 1	0.965	0.955
Orthopedic surgeon 2	0.961	0.948
Orthopedic surgeon 3	0.955	0.956

Orthopedic surgeon 1 and 2 had 3 years of experience. Orthopedic surgeon 3 had 8 years of experience. CNN, convolutional neural network.

**Table 6:** Diagnosis time of orthopedic surgeons with and without CNN model assistance.

	Without CNN model assistance (s)	With CNN model assistance (s)	*p* value
Orthopedic surgeon 1	278.4	162.3	< 0.0001*
Orthopedic surgeon 2	205.2	144.5	< 0.0001*
Orthopedic surgeon 3	233.7	155.5	< 0.0001*

Orthopedic surgeon 1 and 2 had 3 years of experience. Orthopedic surgeon 3 had 8 years of experience. CNN, convolutional neural network.

*p < 0.05.

## Discussion

In the present study, we applied a CNN model to automatically localize and classify fractures on whole-body CT scans of patients from the emergency room with multiple traumas. The CNN model demonstrated good sensitivity in detecting three different types of fractures (pelvic, rib, and spine). In addition, with CNN model assistance orthopedic surgeons showed improved sensitivity for detecting fractures and spent less time to reach a diagnosis, and this was especially true for less experienced orthopedic surgeons.

Here, we applied an object detection algorithm, which is a computer vision task using deep learning, to fracture detection on whole-body CT images of patients with polytrauma. Several previous studies have reported promising results using object detection for specific fractures. Thian et al. trained a CNN model with 7356 radius and ulna fractures on wrist radiographs and reported that the per-image sensitivity was 0.957 for the frontal view and 0.967 for the lateral view; the per-patient sensitivity was 0.981, and the per-image specificity was 0.825 for the frontal view and 0.864 for the lateral view; the per-patient specificity was 0.729^15^. Other studies have also used a CNN model to diagnose rib fractures on CT images^16,19,20^. Kaiume et al.^19^ reported that the sensitivity of the CNN model to detect rib fractures on CT was significantly higher than that of two resident doctors; the sensitivity was 0.645 for the CNN model, 0.313 for resident doctor A, and 0.258 for resident doctor B. Furthermore, Zhou et al.^16^ demonstrated in another CNN model to diagnose rib fractures on CT images that the mean sensitivity was 0.832 (0.861, 0.859, and 0.775 for fresh, healing, and old of rib fractures, respectively) and the mean precision was 0.824 (0.841, 0.857, and 0.774 for fresh, healing, and old of rib fractures, respectively). Additionally, these authors examined whether a CNN model could achieve accurate anatomical localization (right 1st–12th and left 1st–12th ribs) and classification (fresh, healing, and old fractures) of rib fractures, and showed that the sensitivity reached 0.971 and 0.949 on the right and left ribs, respectively^20^. Ukai et al.^17^ proposed an automated method to detect pelvic fractures in the 3D fracture region obtained by integrating multiple 2D fracture candidates, and reported that sensitivity was 0.805 and precision was 0.907. The ability of the CNN model in our study to automatically localize and classify fractures in whole-body CT axis slices of pelvic, rib, and spine fractures was comparable to that of previous CT studies. We found that our CNN model was able to detect and classify pelvic, rib, and spine fractures on whole-body CT with good sensitivity (pelvic 0.839, rib fracture 0.713, spine fracture 0.780). However, the precision for this study was lower than that observed in previous studies. One possible reason to explain this is that the proportion of fractures among the total number of CT axial slices was low (6%). It is known that the precision decreases as prevalence decreases. A second possible reason is that our model frequently misdiagnosed osteophytes and vasculature as new fractures. Nonetheless, previously reported models to automatically localize and classify in CT scans can only detect one specific fracture, the advantage of the model in this study is that it can detect fractures of multiple anatomical parts, including pelvic, rib, and spine fractures on whole-body CT scans with good sensitivity.

With the assistance of a CNN model, orthopedic surgeons can achieve an improved sensitivity for fracture detection and a reduced the time to read and interpret CT scans. In a previous fracture classification study of 480 patients, CNN model assistance for radiographic reading by six types of readers (emergency physicians, orthopedists, radiologists, physician assistants, rheumatologists, family physicians) showed a 10.4% improvement in fracture (thoracolumbar spine, rib cage, hip and pelvis, shoulder and clavicle, elbow and arm, hand and wrist, knee and leg, and foot and ankle) detection sensitivity (0.752 vs 0.648; *p* < 0.001 for superiority) without a reduction in specificity (0.956 vs 0.906, *p* = 0.001 for noninferiority), and the diagnosis time was shortened by an average of 6.3 s per patient with CNN model assistance (*p* = 0.046)^21^. Furthermore, Sato et al. developed a CNN classification model from a relatively large dataset of hip fractures on plain radiographs. The CNN model itself achieved a high diagnostic performance and improved the diagnostic performance of resident doctors (sensitivity of 0.834 without aid to 0.906 with aid *p* < 0.01; accuracy of 0.847 without aid to 0.912 with aid; *p* < 0.01)^22^. In a previous automatic localization and classification study of rib fractures (1079 patients) on CT images, the precision of five radiologists improved from 0.803 to 0.911, the sensitivity increased from 0.624 to 0.863, and the diagnosis time was reduced by an average of 73.9 s with artificial intelligence–assisted diagnosis^16^. In the current study, the sensitivity for orthopedic surgeons improved when assisted by our CNN model (especially for the less experienced orthopedic surgeons), but no reduction in precision was observed. Moreover, CNN model assistance significantly shortened the CT image reading time. This suggests that the deep learning object detection network for pelvic, rib, and spine fractures has good screening ability, as in previous reports.

In a busy emergency department, the great advantage of CNN is that it can serve as a triage system. If the CNN can detect a fracture before orthopedic surgeons or radiologists have a chance to review the images, the suspected fracture image can become a high priority in the worklist. If the physician can prioritize interpretation of images with potentially positive findings, delays in diagnosis can be minimized, thereby improving patient care. Another potential benefit of using CNNs is shorter read times. Even though each examination takes just a few minutes, a reduction in reading time has a significant impact for emergency staff who make multiple clinical decisions each day. Emergency physicians and radiologists on long shifts may experience fatigue and oculomotor strain, resulting in a reduced ability to focus and to detect fractures^23–27^. Fracture recognition using CNN is not only able to detect subtle findings that are difficult for inexperienced physicians to diagnose but also prevents cognitive errors due to human fatigue and biased image interpretation. In summary, application of the CNN model may lead to reductions in missed fractures from whole-body CT images and to faster workflows and improved patient care through efficient diagnosis of polytrauma patients.

The object detection model is not considered an “explainable” type of artificial intelligence, but it does offer some explainability for fracture detection when compared to a classification model that distinguishes between the presence and absence of fractures. Deep learning–based algorithms are often referred to as a “black boxes” because there is no rationale available for their diagnosis. That is, the decision process is not intuitively understood because millions of learned parameters (weights of connections in the network) determine the output of a CNN. This is challenging for clinicians as they tend to make decisions using data acquired with technology that they can understand and trust^28^. Thus, explainability of the algorithm is important in order for users to take informed actions^29^.

We acknowledge that there are several limitations to this study. The first limitation is that we examined only axial slices, not sagittal or coronal images. In the future, the application of 3D object detection models should be considered. Nonetheless, CT images in the axial orientation can be read without reconstruction. Second, this CNN model was validated under conditions different from clinical conditions. It has been validated in a workflow for research purposes and cannot be immediately integrated into the clinical environment. Third, all CT axial slices were read without relevant clinical information. That is, in a clinical setting, emergency physicians and orthopedic surgeons obtain a detailed medical history and examine the patient before interpreting the CT images, which improves the sensitivity and precision of diagnosis. Nevertheless, blinded reading in this study can reflect patients who are unconscious or those whose medical history cannot be fully obtained in the emergency room. Fourth, this study did not investigate fractures of the cervical vertebrae, clavicle, scapula, humerus, sternum, or femur. This is because the proportion of these fractures observed in this study was small. In future work, we will develop a multiplanar object detection model or a 3D object detection model that can diagnose fractures of any anatomical site and validate the model on external dataset.

## Conclusion

We applied a CNN model to the automatic localization and classification of fractures on whole-body CT images from patients in the emergency department with multiple traumas. The CNN model demonstrated good sensitivity in detecting pelvic, spine, or rib fractures. On the other hand, the precision of the CNN model was lower than previous studies. With CNN model assistance, surgeons showed improved sensitivity without loss of precision in detecting fractures and the time of reading and interpreting CT scans was reduced, especially for less experienced orthopedic surgeons. Moreover, the precision reduction was not observed with CNN model assistance.

## Data Availability

The datasets collected and analyzed in this study are not open to the public because they contain information that could compromise the privacy of research participants. However, data are available from the corresponding author upon reasonable request.

## References

[1] Pinto A (2016). Errors in imaging patients in the emergency setting. Br. J. Radiol..

[2] Scaglione M, Iaselli F, Sica G, Feragalli B, Nicola R (2015). Errors in imaging of traumatic injuries. Abdom. Imaging.

[3] Buduhan G, McRitchie DI (2000). Missed injuries in patients with multiple trauma. J. Trauma Inj. Infect. Crit. Care.

[4] Fernholm R (2019). Diagnostic errors reported in primary healthcare and emergency departments: A retrospective and descriptive cohort study of 4830 reported cases of preventable harm in Sweden. Eur. J. Gen. Pract..

[5] Huber-Wagner S (2009). Effect of whole-body CT during trauma resuscitation on survival: A retrospective, multicentre study. Lancet.

[6] Banaste N (2018). Whole-body CT in patients with multiple traumas: Factors leading to missed injury. Radiology.

[7] Rhee PM (2002). Lumbar fractures in adult blunt trauma: Axial and single-slice helical abdominal and pelvic computed tomographic scans versus portable plain films. J. Trauma.

[8] Cho SH, Sung YM, Kim MS (2012). Missed rib fractures on evaluation of initial chest CT for trauma patients: Pattern analysis and diagnostic value of coronal multiplanar reconstruction images with multidetector row CT. Br. J. Radiol..

[9] Gulshan V (2016). Development and validation of a deep learning algorithm for detection of diabetic retinopathy in retinal fundus photographs. JAMA J. Am. Med. Assoc..

[10] Choi JS (2019). Effect of a deep learning framework-based computer-aided diagnosis system on the diagnostic performance of radiologists in differentiating between malignant and benign masses on breast ultrasonography. Korean J. Radiol..

[11] Yamada Y, Maki S, Kishida S, Nagai H (2020). Automated classification of hip fractures using deep convolutional neural networks with orthopedic surgeon-level accuracy: Ensemble decision-making with antero-posterior and lateral radiographs. Acta Orthop.

[12] Maki S (2020). A deep convolutional neural network with performance comparable to radiologists for differentiating between spinal schwannoma and meningioma. Spine (Phila. Pa. 1976).

[13] Tomita N, Cheung YY, Hassanpour S (2018). Deep neural networks for automatic detection of osteoporotic vertebral fractures on CT scans. Comput. Biol. Med..

[14] Suzuki T (2021). Detecting distal radial fractures from wrist radiographs using a deep convolutional neural network with an accuracy comparable to hand orthopedic surgeons. J. Digit. Imaging.

[15] Thian YL (2019). Convolutional neural networks for automated fracture detection and localization on wrist radiographs. Radiol. Artif. Intell..

[16] Zhou QQ (2020). Automatic detection and classification of rib fractures on thoracic CT using convolutional neural network: Accuracy and feasibility. Korean J. Radiol..

[17] Ukai K (2021). Detecting pelvic fracture on 3D-CT using deep convolutional neural networks with multi-orientated slab images. Sci. Rep..

[18] Ren S, He K, Girshick R, Sun J (2017). Faster R-CNN: Towards real-time object detection with region proposal networks. IEEE Trans. Pattern Anal. Mach. Intell..

[19] Kaiume M (2021). Rib fracture detection in computed tomography images using deep convolutional neural networks. Medicine (Baltimore).

[20] Zhou QQ (2022). Precise anatomical localization and classification of rib fractures on CT using a convolutional neural network. Clin. Imaging.

[21] Guermazi A, Tannoury C, Kompel AJ, Murakami AM (2021). Improving radiographic fracture recognition performance and efficiency using artificial intelligence. Radiology.

[22] Sato Y (2021). Artificial intelligence improves the accuracy of residents in the diagnosis of hip fractures: A multicenter study. BMC Musculoskelet. Disord..

[23] Krupinski EA, Berbaum KS, Caldwell RT, Schartz KM, Kim J (2010). Long radiology workdays reduce detection and accommodation accuracy. J. Am. Coll. Radiol..

[24] Reiner BI, Krupinski E (2012). The insidious problem of fatigue in medical imaging practice. J. Digit. Imaging.

[25] Gaba DM, Howard SK (2002). Fatigue among clinicians and the safety of patients. N. Engl. J. Med..

[26] Lee CS, Nagy PG, Weaver SJ, Newman-Toker DE (2013). Cognitive and system factors contributing to diagnostic errors in radiology. Am. J. Roentgenol..

[27] Hartigan S (2020). Review of the basics of cognitive error in emergency medicine: Still no easy answers. West. J. Emerg. Med..

[28] Meskó B, Görög M (2020). A short guide for medical professionals in the era of artificial intelligence. npj Digit. Med..

[29] Langlotz CP (2019). A roadmap for foundational research on artificial intelligence in medical imaging: From the 2018 NIH/RSNA/ACR/The academy workshop. Radiology.

